# Depressive symptoms in cognitively unimpaired older adults are associated with lower structural and functional integrity in a frontolimbic network

**DOI:** 10.1038/s41380-022-01772-8

**Published:** 2022-10-18

**Authors:** Edelweiss Touron, Inès Moulinet, Elizabeth Kuhn, Siya Sherif, Valentin Ourry, Brigitte Landeau, Florence Mézenge, Denis Vivien, Olga M. Klimecki, Géraldine Poisnel, Natalie L. Marchant, Gaël Chételat, for the Alzheimer’s Disease Neuroimaging Initiative, the Medit-Ageing Research Group, Eider M. Arenaza-Urquijo, Florence Allais, Claire André, Julien Asselineau, Sebastian Baez Lugo, Martine Batchelor, Axel Beaugonin, Alexandre Bejanin, Pierre Champetier, Anne Chocat, Fabienne Collette, Sophie Dautricourt, Eglantine Ferrand-Devouge, Robin De Flores, Vincent De La Sayette, Pascal Delamillieure, Marion Delarue, Yacila I. Deza-Araujo, Hélène Esperou, Francesca Felisatti, Eric Frison, Francis Gheysen, Julie Gonneaud, Marc Heidmann, Thien Huong Tran, Frank Jessen, Pierre Krolak-Salmon, Gwendoline Le Du, Valérie Lefranc, Antoine Lutz, Jose-Luis Molinuevo, Cassandre Palix, Léo Paly, Géraldine Rauchs, Stéphane Réhel, Florence Requier, Eric Salmon, Raquel Sanchez, Corinne Schimmer, Matthieu Vanhoutte, Patrik Vuilleumier, Caitlin Ware, Miranka Wirth

**Affiliations:** 1 Unité 1237 PhIND “Physiopathology and Imaging of Neurological Disorders”, Institut National de la Santé et de la Recherche Médicale, Blood and Brain @ Caen-Normandie, GIP Cyceron, Normandie Université, Université de Caen, Caen, France; 2 Unité 1077 NIMH “Neuropsychologie et Imagerie de la Mémoire Humaine,” Institut National de la Santé et de la Recherche Médicale, Normandie Université, Université de Caen, PSL Université, EPHE, CHU de Caen-Normandie, GIP Cyceron, Caen, France; 3 Département de Recherche Clinique, CHU de Caen-Normandie, Caen, France; 4 Clinical Psychology and Behavioral Neuroscience, Faculty of Psychology, Technische Universität Dresden, 01187 Dresden, Germany; 5 Division of Psychiatry, University College London, London, UK; 6 EUCLID/F-CRIN Clinical Trials Platform, Bordeaux, France; 7 Swiss Center for Affective Sciences, Department of Medicine, University of Geneva, Geneva, Switzerland; 8 Independent Meditation Teacher, Caen, France; 9 GIGA–Cyclotron Research Centre, In Vivo Imaging and Psychology and Cognitive Neuroscience Unit, Liège University, Liège, Belgium; 10 Service de Neurologie, CHU de Caen, Caen, France; 11 Département de Psychiatrie, Centre Hospitalier Universitaire de Caen, Caen, France; 12 Institut National de la Santé et de la Recherche Médicale, Pôle de Recherche Clinique, 75013 Paris, France; 13 Lyon Neuroscience Research Center, Institut National de la Santé et de la Recherche Médicale Unité 1028, Centre National de la Recherche Scientifique Unité Mixte de Recherche 5292, Lyon University, Lyon, France; 14 Uniklinik Köln, Cologne, Germany; 15 Hospices Civils de Lyon, Lyon, France; 16 Institut d’Investigacions Biomèdiques August Pi i Sunyer, Barcelona, Spain; 17 Université de Caen, Caen, France; 18 Deutsches Zentrum für Neurodegenerative Erkrankungen, Dresden, Germany

**Keywords:** Neuroscience, Biomarkers, Depression

## Abstract

Subclinical depressive symptoms are associated with increased risk of Alzheimer’s disease (AD), but the brain mechanisms underlying this relationship are still unclear. We aimed to provide a comprehensive overview of the brain substrates of subclinical depressive symptoms in cognitively unimpaired older adults using complementary multimodal neuroimaging data. We included cognitively unimpaired older adults from the baseline data of the primary cohort Age-Well (*n* = 135), and from the replication cohort ADNI (*n* = 252). In both cohorts, subclinical depressive symptoms were assessed using the 15-item version of the Geriatric Depression Scale; based on this scale, participants were classified as having depressive symptoms (>0) or not (0). Voxel-wise between-group comparisons were performed to highlight differences in gray matter volume, glucose metabolism and amyloid deposition; as well as white matter integrity (only available in Age-Well). Age-Well participants with subclinical depressive symptoms had lower gray matter volume in the hippocampus and lower white matter integrity in the fornix and the posterior parts of the cingulum and corpus callosum, compared to participants without symptoms. Hippocampal atrophy was recovered in ADNI, where participants with subclinical depressive symptoms also showed glucose hypometabolism in the hippocampus, amygdala, precuneus/posterior cingulate cortex, medial and dorsolateral prefrontal cortex, insula, and temporoparietal cortex. Subclinical depressive symptoms were not associated with brain amyloid deposition in either cohort. Subclinical depressive symptoms in ageing are linked with neurodegeneration biomarkers in the frontolimbic network including brain areas particularly sensitive to AD. The relationship between depressive symptoms and AD may be partly underpinned by neurodegeneration in common brain regions.

## Introduction

Late-life depression appears as one of the main potentially modifiable late-life risk factors for dementia [1], and it has been projected that a significant number of Alzheimer’s disease (AD) cases could be prevented if depression is reduced throughout life [2]. Even subclinical depressive symptoms—that do not meet diagnostic criteria for clinical depressive disorder—worsen quality of life and health in older adults, increasing disability, morbidity and mortality [3, 4]. They are also associated with increased risk for both clinical depressive disorder and AD. Thus, subclinical depressive symptoms are associated with higher risk of cognitive decline in cognitively unimpaired (CU) older adults, with each additional symptom increasing the risk of AD by about 20% [5, 6]. Moreover, depressive symptoms are frequent in patients with mild cognitive impairment (MCI) or dementia, and they increase the risk of progression to AD dementia in MCI patients [7, 8]. However, the mechanisms linking depressive symptoms and AD risk are still unclear. Neuroimaging studies investigating the brain changes related to depressive symptoms in ageing may help to better understand these mechanisms underlying this relationship. Studying the preclinical stages of these states, namely, subclinical depressive symptoms and AD biomarkers in CU older adults, could help disentangle their possible interactions as they develop.

Previous magnetic resonance imaging (MRI) studies in CU older adults have reported that subclinical depressive symptoms were associated with lower gray matter (GM) volume or cortical thickness mainly in the medial prefrontal cortex and temporal regions including the hippocampus [9–17]. Studies with positron emission tomography (PET) are sparse; they suggest that depressive symptoms are associated with lower glucose metabolism or perfusion in AD-related brain regions such as the precuneus and posterior cingulate cortex, and in fronto-temporal regions including the hippocampus [12, 18–20]. Regarding amyloid-PET studies, findings are mixed, with some studies reporting an association between depressive symptoms and higher amyloid deposition both cross-sectionally and longitudinally [21–24], while others did not find such a relationship [25, 26].

Most of the previous studies used regions-of-interest (ROI)-based analyses, which did not allow for a global picture of the brain changes associated with subclinical depressive symptoms. Moreover, a majority of studies included only one imaging modality, most often focusing on changes in GM volume. Our main goal with this study was to provide a more comprehensive overview of the brain substrates of subclinical depressive symptoms in CU older adults using whole brain voxel-wise analyses with structural, functional and molecular neuroimaging data in two independent cohorts. With this approach, we aim to offer a better understanding of the early brain mechanisms underlying the links between subclinical depressive symptoms in ageing and the preclinical stage of AD, which are both associated with increased risk of developing clinical stages of these states. We hypothesized that the presence of subclinical depressive symptoms would be associated with brain structural and functional alterations related to late-life depression and AD—especially in frontolimbic regions—as well as with higher neocortical amyloid deposition.

## Materials and methods

### Study design

Data from CU older adults from two independent protocols were selected. The analyses were first conducted in the primary cohort Age-Well (monocentric study) and replicated in a larger cohort with the Alzheimer’s disease Neuroimaging Initiative data (ADNI; multicentric study).

### Participants

#### Age-Well cohort

One hundred thirty-five CU older adults were included from the baseline visit of the Age-Well randomized controlled trial of the Medit-Ageing European project [27], sponsored by the French National Institute of Health and Medical Research (INSERM). Participants were recruited from the general population with the main following eligibility criteria: native French speaker, aged at least 65 years, retired for at least 1 year, educated for at least 7 years and showing performance within the normal range for age and educational level on standardized cognitive tests (see Tables 1 and 2 in [27] for details). Participants had no evidence of a major neurological or psychiatric disorder, chronic disease or acute unstable illness, no history of cerebrovascular disease, and no current or recent medication that may interfere with cognitive functioning (including antidepressants and anxiolytics). Notably, the absence of major depression was assessed using a clinician-administered questionnaire, the Montgomery-Åsberg Depression Rating Scale (MADRS) [28], with a cut-off value of 6 (participants with MADRS > 6 were excluded). All participants gave their written informed consent prior to the examinations, and the Age-Well randomized clinical trial was approved by the ethics committee (Comité de Protection des Personnes Nord-Ouest III, Caen, France; trial registration number: EudraCT: 2016-002441-36; IDRCB: 2016-A01767-44; ClinicalTrials.gov Identifier: NCT02977819).

#### ADNI cohort

Two hundred fifty-two CU older adults from ADNI were included in our study as a replication cohort. The main exclusion criteria included the presence of psychiatric illness (major depression or bipolar disorder) or neurological disease (see [29] for details). The absence of major depression was assessed using the self-reported Geriatric Depression Scale (GDS) [30], and the investigators indicated that “although differing sensitivities and specificities have been obtained across studies, a score >5 was considered suggestive of depression and warranted a follow-up interview” (in our study, only one ADNI participant was concerned with a GDS score = 6) and “scores >10 are almost always depression”. Details regarding the cohort recruitment and all data collection methods is available online (http://adni.loni.usc.edu/). Only participants with an available score of depressive symptoms and whose multimodal neuroimaging scans were acquired no more than 3 months after the assessment of depressive symptoms were selected. The ADNI study was approved by the institutional review boards of all of the participating institutions. Informed written consent was obtained from all participants at each site.

### Assessment of subclinical depressive symptoms and classification of participants

In this study we were specifically interested in the presence of subclinical depressive symptoms, which was assessed using the 15-item version of the GDS. This self-reported questionnaire ranges from 0 to 15, and a higher score reflects the presence of more depressive symptoms [30, 31]. Given that all participants were screened for the lack of depression, GDS scores were rather low showing a floor-effect and a non-linear distribution. Therefore, instead of the severity of the symptoms (which lacked variability) we were primarily interested in comparing participants with or without subclinical depressive symptoms. Thus, based on the GDS, participants were classified as having subclinical depressive symptoms (DepS; GDS > 0) or not (NoDepS; GDS = 0). This threshold was selected as, while there is no consensus to date in the current literature to define “subclinical” depressive symptoms [32], previous studies have shown that each additional depressive symptom (including 1-point increase in 15-item GDS) significantly increases the risk of AD in CU older adults [5, 33]. In Age-Well, the DepS and NoDepS groups included 77 and 58 participants respectively. In ADNI, the DepS and NoDepS groups included 134 and 118 participants respectively. Additional analyses were also performed with the severity (number) of subclinical depressive symptoms, for the sake of completeness.

### Assessment of cognitive performance

Global cognition was measured using the Mini-Mental State Examination (MMSE [34], scores from 0 to 30) within each cohort, as well as using the Mattis Dementia Rating Scale (DRS [35], scores from 0 to 144) in Age-Well, and the Montreal Cognitive Assessment (MoCA [36], scores from 0 to 30) in ADNI. Verbal episodic memory was assessed using the immediate free recall subscore of the California Verbal Learning Test (sum of scores from the five trials of the 16-word list) (CVLT [37], scores from 0 to 80) in Age-Well and the Immediate subscore of the Rey Auditory Verbal Learning Test (sum of scores from the five trials of the 15-word list) (RAVLT [38], scores from 0 to 75) in ADNI. The MoCA and RAVLT scores were not available for 5 and 3 participants, respectively.

### Neuroimaging procedure

#### Age-Well cohort

All participants were scanned on the same MRI scanner (Philips Achieva; 3.0 T) and PET camera (Discovery RX VCT 64 PET-CT; General Electric Healthcare) at the Cyceron Center (Caen, France). High-resolution T1-weighted structural imaging were acquired to measure GM volume and an echo-planar imaging/spin echo diffusion weighted sequence (DKI) was performed to obtain white matter (WM) microstructural integrity measurements. Mean kurtosis parameter maps reflected WM microstructural integrity based on the number, density, orientation, and degree of organization of WM microstructures [39]. Myelin and axonal integrity was also estimated from the radial and axial parameters of DKI, respectively [40]. Fluorine 18-labeled (^18^F) florbetapir-PET scans were obtained with a 10-min acquisition beginning 50 min after the intravenous injection reflecting amyloid burden. ^18^F-fluorodeoxyglucose (FDG)-PET scans were acquired on a subset of participants (*n* = 92) to measure brain glucose metabolism. The detailed acquisition and preprocessing procedures [27] are available in Supplementary Material 1. The sample size for each imaging modality is reported in Supplementary Table 1.

#### ADNI cohort

Acquisition processes of structural MRI, FDG- and florbetapir-PET imaging are described at http://adni.loni.usc.edu/data-samples/data-types/. DKI images were not available. The same preprocessing procedures as in Age-Well were applied on ADNI data, except that the segmentation of the MRI scans was only based on the T1-weighted images in ADNI (while both the T1 and FLAIR were used in Age-Well).

### Statistical analyses

#### Between-group comparisons

Between-group differences for demographic, cognitive, and psychoaffective variables were assessed both within and across cohorts, using Student’s *t* tests for continuous variables and *χ*^2^ tests for categorical variables with statistical significance set to *p* < 0.05.

Voxel-wise group differences in GM volume, WM integrity, glucose metabolism and amyloid burden were explored using analyses of covariance (ANCOVA) in SPM12. In both cohorts, all voxel-wise analyses were adjusted for age, sex and education, as well as self-reported anxiety symptoms when available (only for Age-Well). Results were evaluated for significance at *p*_uncorrected_ < 0.005 combined with a minimum cluster size determined by Monte–Carlo simulations using the AFNI’s 3dClustSim program to achieve a corrected statistical significance of *p* < 0.05.

## Results

### Participants’ characteristics

Demographic data, cognitive performance and psychoaffective symptoms for each group in both cohorts, as well as between-group differences, are reported in Table 1.

**Table 1:** Participants’ characteristics and between-group comparisons within and between cohorts.

Age-Well—Primary cohort (*N* = 135)	NoDepS group	DepS group	Between-group comparisons		
			*p* value	*t* or *χ*² value	Mean difference [95% CI]
*N* (%)	58 (43)	77 (57)			
Demographic data					
Gender: Female *N* (%)	28 (48.27)	55 (71.43)	**0.01**	**6.54**	
Age, years (range)	69.41 ± 3.91 (64–83)	68.45 ± 3.63 (65–79)	0.14	−1.47	−0.96 [−2.25–0.33]
Education, years (range)	13.12 ± 3.17 (7–22)	13.18 ± 3.04 (7–20)	0.91	0.11	0.06 [−1.00–1.13]
Florbetapir SUVR (range)	0.96 ± 0.21 (0.73–1.76)	0.98 ± 0.20 (0.72–1.73)	0.47	0.72	0.03 [−0.05–0.10]
Amyloid positive *N* (%)	10 (17.24)	20 (26.31)	0.30	1.08	
APOEε4 carriers *N* (%)	15 (25.86)	21 (27.27)	1.00	<0.001	
Cognition measures					
MMSE (range)	28.93 ± 0.95 (26–30)	29.11 ± 1.09 (26–30)	0.34	0.97	0.17 [−0.18–0.53]
DRS (range)	140.90 ± 2.79 (130–144)	141.03 ± 2.56 (133–144)	0.78	0.28	0.13 [−0.78–1.04]
CVLT Immediate (range)	57.36 ± 8.61 (30–71)	57.69 ± 7.70 (37–73)	0.82	0.23	0.33 [−2.46–3.11]
Psychoaffective variables					
GDS (range)	0.00 ± 0.00 (0–0)	2.25 ± 1.77 (1–11)	**<0.001**	**9.65**	**2.25 [1.79–2.71]**
STAI-B (range)	31.60 ± 6.18 (20–51)	36.82 ± 6.77 (24–54)	**<0.001**	**4.59**	**5.21 [2.97–7.46]**

ADNI—Replication cohort (*N* = 252)	NoDepS group	DepS group	Between-group comparisons		
			*p* value	*t* or *χ*² value	Mean difference [95% CI]
*N* (%)	118 (47)	134 (53)			
Demographic data					
Gender: Female *N* (%)	65 (55.08)	78 (58.20)	0.71	0.14	
Age, years (range)	73.35 ± 5.69^a^ (63–85)	73.66 ± 6.32^a^ (59–95)	0.68	0.41	0.31 [−1.19–1.81]
Education, years (range)	16.50 ± 2.59^a^ (8–20)	16.71 ± 2.35^a^ (12–20)	0.50	0.67	0.21 [−0.40–0.82]
Florbetapir SUVR (range)	1.21 ± 0.37^a^ (0.76–2.55)	1.13 ± 0.36^a^ (0.74–3.24)	0.11	−1.60	−0.07 [−0.17–0.02]
Amyloid positive *N* (%)	69 (58.47)^a^	63 (47.01)^a^	0.09	2.86	
APOEε4 carriers *N* (%)	39 (33.05)	33 (24.63)	0.11	2.58	
Cognition measures					
MMSE (range)	28.99 ± 1.16 (25–30)	28.96 ± 1.26 (24–30)	0.81	−0.24	−0.04 [−0.34–0.27]
MoCA (range)	25.67 ± 2.25 (20–30)	25.63 ± 2.47 (19–30)	0.89	−0.14	−0.04 [−0.64–0.56]
RAVLT Immediate (range)	46.08 ± 10.16 (20–70)	45.56 ± 10.21 (23–69)	0.71	0.37	0.48 [−2.07–3.03]
Psychoaffective variables					
GDS (range)	0.00 ± 0.00 (0–0)	1.78 ± 1.11^a^ (1–6)	**<0.001**	**17.41**	**1.78 [1.58–1.98]**
STAI-B (range)	NC	NC			

Data are presented as mean ± standard deviation of participants unless otherwise indicated.

Between-group differences in each cohort were assessed using Student’s *t* tests for continuous variables and *χ*^2^ tests for categorical variables. Statistical significance was set to *p* < 0.05 for all analyses.

*N* sample size, *NoDepS* group without depressive symptoms, *DepS* group with subclinical depressive symptoms, *SUVR* standard uptake value ratio, *MMSE* Mini-Mental State Examination, *DRS* Mattis Dementia Rating Scale, *CVLT* California Verbal Learning Test, *GDS* Geriatric Depression Scale, *STAI-B* State-Trait Anxiety Inventory form Y-B, *MoCA* Montreal Cognitive Assessment, *RAVLT* Rey Auditory Verbal Learning Test, *NC* not collected.

^a^Significant between-group differences between cohorts (see details in Supplementary Table 2).

Bold values indicate statistical significance *p* < 0.05.

In Age-Well, participants with depressive symptoms had a higher proportion of women and a higher score of anxiety symptoms than those without symptoms. The two groups did not differ in any other demographic or cognitive variables. There was no difference between participants with versus without depressive symptoms in ADNI.

When comparing groups between cohorts, the proportion of participants classified as having subclinical depressive symptoms was similar between cohorts (*χ*^2^ = 0.39 *p* = 0.54). However, Age-Well participants were younger, less educated, and had lower florbetapir SUVR and proportion of amyloid-positive individuals than ADNI participants. Moreover, those with depressive symptoms in Age-Well had a higher GDS score.

### Brain changes associated with the presence of subclinical depressive symptoms

#### Gray matter volume

**Fig. 1:**
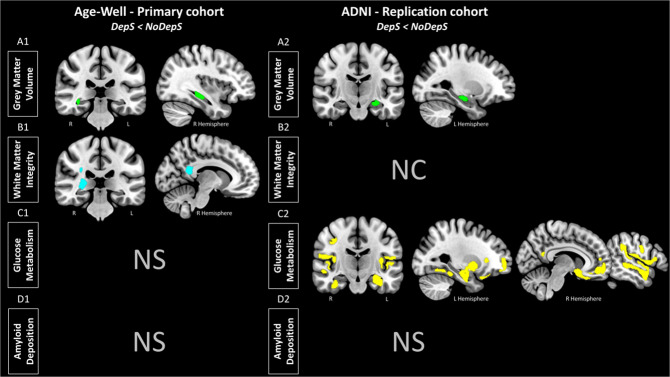
Brain substrates of subclinical depressive symptoms in cognitively unimpaired older adults. Results of the voxel-wise between-group differences in gray matter volume (**A**, green), white matter integrity (mean kurtosis) (**B**, blue), glucose metabolism (**C**, yellow) and amyloid deposition (**D**) in Age-Well (left panel) and ADNI (right panel). Analyses were adjusted for age, sex and education. Anxiety symptoms were added as a covariate in Age-Well. All results are presented at a *p*_uncorrected_ < 0.005 threshold combined with a cluster-level multiple comparisons correction. DepS group with subclinical depressive symptoms, NoDepS group without depressive symptoms, NS not significant, NC not collected, R right, L left.

Participants with subclinical depressive symptoms showed lower GM volume compared with participants without symptoms in the right hippocampus in Age-Well (Fig. 1A1), and in the left hippocampus in ADNI (Fig. 1A2). Peak statistics and coordinates of significant clusters are detailed in Supplementary Table 3. In both cohorts, the volume of the contralateral hippocampus was also found when using a more permissive threshold (i.e., at *p*_uncorrected_ < 0.005 in Age-Well and at *p*_uncorrected_ < 0.01 in ADNI).

#### White matter integrity

In Age-Well, participants with subclinical depressive symptoms showed lower WM microstructural integrity (mean kurtosis) than participants without depressive symptoms mainly in the posterior cingulum and corpus callosum (splenium part), fornix and inferior longitudinal fasciculus (Fig. 1B1). They also showed lower myelin integrity (radial kurtosis) in the posterior cingulum and corpus callosum (splenium part) (Supplementary Fig. 1). No significant differences were observed regarding the axonal integrity of the WM (axonal kurtosis).

#### Glucose metabolism

While in Age-Well, no significant difference in glucose metabolism was observed between the two groups, in ADNI, participants with subclinical depressive symptoms showed lower glucose metabolism mainly in the medial temporal lobe, temporal cortex, and medial and dorsolateral prefrontal, anterior cingulate, temporoparietal, precuneus/posterior cingulate cortices, and insula (Fig. 1C2).

#### Amyloid deposition

No difference in brain amyloid deposition was observed between participants with depressive symptoms versus those without symptoms in either Age-Well or ADNI.

### Additional analyses

#### Neuroimaging measures

Neuroimaging analyses were replicated without partial volume effects (PVE) correction for PET images. Results were similar with differences in cluster sizes for glucose metabolism analyses in ADNI, as illustrated in Supplementary Fig 2. Neuroimaging results were also similar when adding the MMSE as a covariate (data not shown).

**Fig. 2:**
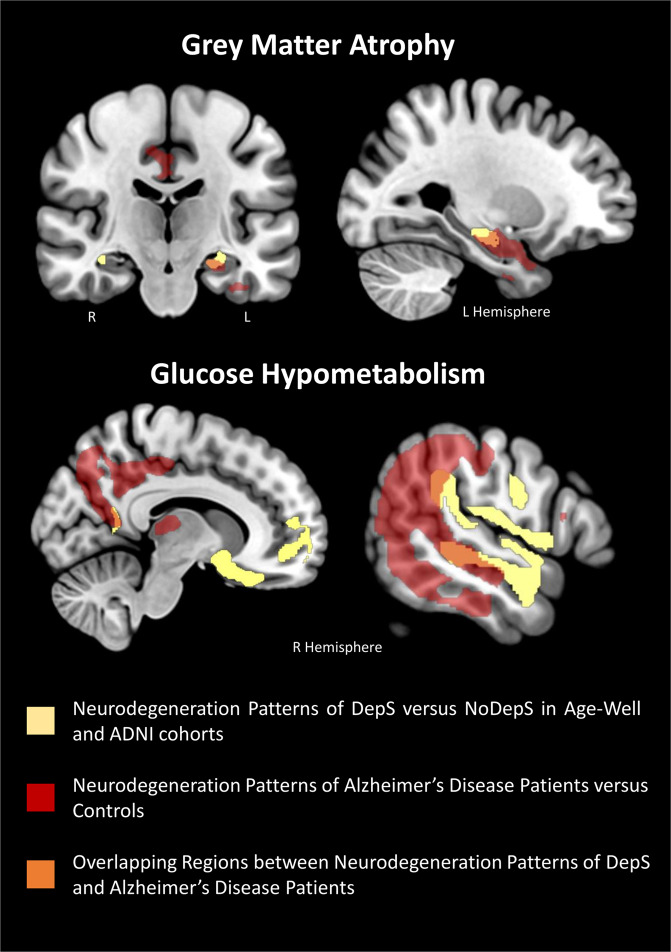
Illustration of the overlap between the brain substrates of subclinical depressive symptoms and the pattern of neurodegeneration in Alzheimer’s disease (AD). The patterns of gray matter atrophy (at the top) and hypometabolism (at the bottom) in participants with subclinical depressive symptoms compared to those without are represented in yellow. They are overlapped on the respective patterns of gray matter atrophy (at the top) and hypometabolism (at the bottom) typically found in patients with AD and obtained here by comparing a group of 56 cognitively impaired amyloid-positive patients on the Alzheimer’s continuum to 28 controls from the IMAP + cohort ([41]; see Supplementary Material 2 for details). The superimposition allows areas of overlap to be highlighted (in orange), notably in the hippocampus, precuneus, posterior cingulate—retrosplenial area and temporoparietal region. DepS group with subclinical depressive symptoms, NoDepS group without depressive symptoms, R right, L left.

To highlight the overlap between the brain substrates of subclinical depressive symptoms from both cohorts and the pattern of neurodegeneration typically found in patients with AD, we superimposed our findings over patterns of GM atrophy and hypometabolism from a group of 56 cognitively impaired amyloid-positive patients on the Alzheimer’s continuum compared to 28 controls from the independent Imagerie Multimodale de la maladie d’Alzheimer à un stade Précoce (IMAP+) cohort ([41]; see the Supplementary Material 2 for details). The superimposition of both patterns overlapped notably in the hippocampus, precuneus, posterior cingulate—retrosplenial area and temporoparietal region, as illustrated in Fig. 2.

We then aimed to further assess whether the severity of subclinical depressive symptoms was correlated with the brain changes found in the main neuroimaging analyses. We extracted the mean value within the clusters previously highlighted in the voxel-wise between-group comparison analyses (i.e., GM volume and WM integrity in Age-Well, as well as GM volume and glucose metabolism in ADNI) on the corresponding non-smoothed MRI and PET images for each participant. Given that all participants were screened for the lack of depression, GDS scores were rather low showing a floor-effect and a non-linear distribution. Therefore, we performed non-parametric analyses using Spearman’s partial correlations to assess the associations between the severity of subclinical depressive symptoms and the extracted neuroimaging measures within the group of participants with subclinical depressive symptoms, as well as within the entire sample, for both cohorts. Analyses were corrected for age, sex and education, as well as anxiety symptoms (only for Age-Well).

We found that higher subclinical depressive symptoms were associated with lower GM volume (rho = −0.211 *p* = 0.016 in Age-Well and rho = −0.251 *p* < 0.001 in ADNI), WM integrity (rho = −0.232 *p* = 0.008 for mean kurtosis and rho = −0.236 *p* = 0.007 for radial kurtosis) and glucose metabolism (rho = −0.277 *p* < 0.001) in the clusters of interest within the entire samples, for both cohorts (Supplementary Table 4). These associations were not significant within the group of participants with subclinical depressive symptoms in either Age-Well or ADNI.

#### Psychoaffective measures

We sought to better characterize the psychoaffective difficulties experienced by participants with subclinical depressive symptoms compared to those without depressive symptoms in Age-Well (assessments not available in ADNI). For this purpose, we investigated between-group differences in self-report of positive and negative affect (i.e., positive and negative emotions or feelings) based on the Positive and Negative Affect Schedule (PANAS [42]), ruminative brooding (i.e., repetitive passive and judgmental thoughts about one’s mood) based on the Rumination Response Scale (RRS [43], defusion (i.e., ability to achieve psychological distance from one’s thoughts and feelings) based on the Drexel Defusion Scale (DDS [44]) and emotion regulation abilities (i.e., ability to regulate one’s emotions through cognitive reappraisal and/or expressive suppression strategies) based on the Emotion Regulation Questionnaire (ERQ [45]) (see Supplementary Material 3 for details). We used ANCOVAs adjusted for age, sex and education. Participants with subclinical depressive symptoms showed higher negative affect (*F* = 5.82 *p* = 0.017) and ruminative brooding (*F* = 4.32 *p* = 0.040), as well as lower psychological defusion (*F* = 9.33 *p* = 0.003) than those without symptoms (Supplementary Table 5). No significant differences were observed regarding positive affect and emotion regulation abilities.

## Discussion

The aim of this study was to provide a comprehensive overview of the brain substrates of subclinical depressive symptoms in CU older adults using complementary multimodal neuroimaging data in two independent cohorts. We showed that participants with subclinical depressive symptoms from both cohorts presented lower GM volume in the hippocampus. In ADNI, glucose hypometabolism was also found in the hippocampus and extended to the amygdala, precuneus/posterior cingulate cortex, medial and dorsolateral prefrontal cortex, insula, and temporoparietal cortex. In Age-Well, we also found lower WM integrity mainly in the fornix, posterior cingulum and corpus callosum and inferior longitudinal fasciculus. Furthermore, the presence of subclinical depressive symptoms was not associated with brain amyloid deposition in either cohort.

### Subclinical depressive symptoms are consistently associated with neurodegeneration biomarkers in the hippocampus

The association between subclinical depressive symptoms and lower GM volume in the hippocampus in CU older adults is in line with previous studies using both group comparisons or correlation analyses—cross-sectionally and longitudinally [10, 12–14, 16, 17, 46–49]. Interestingly, lower glucose metabolism colocalized with lower GM volume in the hippocampus in ADNI, which corroborates a previous FDG-PET study using a ROI-based approach [12]. This finding was not recovered in Age-Well but this might reflect a lack of power related to the smaller sample size (*n* = 92, against *n* = 252 in ADNI). Our findings thus suggest that the hippocampus is particularly sensitive to subclinical depressive symptoms, as it is to AD, being the main target of structural and functional neurodegeneration in both conditions [50, 51]. Thus, we assume that these common alterations in the hippocampus could partly underlie the link between subclinical depressive symptoms, increased risk of clinical depressive disorder, and AD. Potential mechanisms mediating the association between subclinical depressive symptoms and hippocampal neurodegeneration could involve cortisol neurotoxicity, neuroinflammation and/or preclinical AD tau aggregates. In late-life depression, cortisol-mediated hippocampal neurotoxicity has been proposed as a main etiological mechanism [52], with the hippocampus being particularly vulnerable to high levels of cortisol, resulting in neuronal death and/or suppressed neurogenesis [52, 53]. Neuroinflammatory processes may also be involved, as increased inflammatory cytokine levels was reported in older adults with subclinical or clinical depressive disorders, and elevated cytokines levels may impair hippocampal neurogenesis—leading to GM volume loss [54]. Another mechanism could involve tau aggregates in the hippocampus, as increased tau accumulation has been associated with depressive disorders, and the hippocampus is one of the first regions to show tau pathology in preclinical AD [51, 55, 56].

### Brain substrates of subclinical depressive symptoms extend beyond the hippocampus to a frontolimbic network

In ADNI, the pattern of glucose metabolism associated with subclinical depressive symptoms extended beyond the hippocampus to the precuneus/posterior cingulate, temporoparietal and medial prefrontal cortex—known to be vulnerable to metabolic changes in AD [51]. Our results are consistent with the few PET studies in the field reporting lower glucose metabolism or perfusion associated with depressive symptoms in the precuneus, posterior cingulate and fronto-temporal regions [18–20]. Here, the presence of subclinical depressive symptoms was also associated with lower WM integrity mainly in the fornix, posterior cingulum and corpus callosum, and inferior longitudinal fasciculus. These results are in line with previous studies in CU older adults showing an association between subclinical depressive symptoms and reduced WM integrity in the corpus callosum and inferior longitudinal fasciculus [57–59] as well as in frontal regions and in the global WM [9, 11, 60]. Similar links were reported in late-life depression, especially in the cingulum, corpus callosum, but also in the uncinate fasciculus and frontal lobe [61]. Furthermore, the results we obtained with the radial and axonal parameters of DKI suggest that these WM alterations rather reflect demyelination processes than axonal degeneration. This demyelination may result from neuroinflammation; as mentioned above, depressive disorders have been related to high level of inflammatory markers which may alter myelin sheaths [54, 62]. Overall, our findings highlight that the presence of subclinical depressive symptoms is associated with lower integrity of WM microstructure in regions similar to those found to be associated with clinical depressive disorder in ageing.

Interestingly, the fornix and the posterior cingulum and corpus callosum are also among the WM tracts the most altered in early AD stages [63, 64]. Moreover these tracts are structurally connected to the brain regions found to be associated with subclinical depressive symptoms in our study—including specific AD biomarkers such as hippocampal atrophy and posterior cingulate hypometabolism [65, 66]. Previous work highlighted that cingulum alteration was related to hippocampal atrophy and posterior cingulate hypometabolism in MCI patients [67]. Thus, it is possible that the functional alterations notably observed with FDG-PET in older adults with depressive symptoms are related to disconnection from the atrophied hippocampus associated with the disruption of the connecting WM tracts, as proposed in AD [67].

### Subclinical depressive symptoms are associated with neurodegeneration in large brain networks involved in emotion regulation, self-referential processes and memory

Most of the structures associated with subclinical depressive symptoms, namely the hippocampus, amygdala, cingulum, fornix, insula, medial and dorsolateral prefrontal cortex, are key components of the limbic/paralimbic and frontal brain networks—referred to as the frontolimbic network here [68, 69]. This network is mainly involved in emotional and mood processes, including identification of emotional stimuli, generation and/or regulation of the affective state and emotional behavior, as well as memory processes [69]. In line with these findings, participants with subclinical depressive symptoms showed higher negative affect (i.e., negative emotions and feelings) than those without depressive symptoms in Age-Well. Moreover, in late-life depression the frontolimbic network is also particularly impaired and its dysfunction may contribute to the severity of the symptoms [70, 71]. Interestingly, when considering depressive symptoms as a continuous variable, we also found that subclinical depressive symptom severity was associated with levels of brain alterations reported above in the entire samples, for both cohorts. The brain substrates of subclinical depressive symptoms also involve regions of the default mode network (DMN), including the precuneus/cingulate posterior, medial prefrontal, temporoparietal and temporal cortex and the hippocampus. This network is involved in self-referential processes and memory. Changes in the activity and functional connectivity of this network have been observed in late-life depression, and are thought to be related to a dysregulation of mental content in favor of negative thoughts and rumination [72–75]. Our findings complement this literature by showing that participants with subclinical depressive symptoms also exhibited mental regulation difficulties, with higher ruminative brooding (i.e., repetitive passive and judgmental thoughts about one’s mood) and lower defusion (i.e., lower ability to achieve psychological distance from one’s thoughts and feelings) in Age-Well. Furthermore, the DMN includes the regions the most sensitive to AD, as neurodegenerative changes and amyloid deposition are mainly located in this network [76–78]. The insula, dorsolateral prefrontal cortex and precuneus are also part of the salience and cognitive control networks [79, 80]. The salience network is involved in assessing the relevance of stimuli and events, and its dysfunction in the case of depressive disorders—associated with aberrant switching between the DMN and the cognitive control network—may contribute to patients’ difficulties in disengaging self-focus processes involving negatively biased thoughts [79, 81]. The cognitive control network is more involved in high-level cognitive processes, including executive functions such as attention, planning or working memory to achieve a specific goal; functional disruption in this network is thought to reflect decreased cognitive control of attention and emotion regulation [80]. Thus, the brain substrates related to subclinical depressive symptoms overlap in several interconnected brain networks; these alterations may contribute to subsequent network brain dysfunctions as described in clinical depressive disorder and AD, which partly underly the core symptoms of these diseases.

### Subclinical depressive symptoms and AD neuroimaging biomarkers

Although the brain substrates of subclinical depressive symptoms partly overlap with AD neurodegeneration biomarkers, they were not associated with brain amyloid deposition in either cohort. This is consistent with most [22–25], but not all [21] previous cross-sectional studies. Longitudinal studies led to more consistent findings, showing that higher baseline level of depressive symptoms was related to increased amyloid deposition over time. The opposite direction was also observed with higher baseline amyloid deposition associated with higher level of depressive symptoms over time [22–24]. Thus, the links between subclinical depressive symptoms and AD may not initially involve amyloid deposition but rather neurodegeneration in partially common brain regions, suggesting that subclinical depressive symptoms may be a risk factor for AD rather than a prodromal manifestation of the disease.

### Strengths and limitations

The main strength of our study was to provide an overview of the brain substrates of subclinical depressive symptoms in CU older adults using complementary multimodal neuroimaging data (i.e., GM volume, WM integrity, glucose metabolism, and amyloid deposition). Furthermore, the results were partly replicated in two independent cohorts with large sample sizes (i.e., Age-Well and ADNI)—using the same scale to assess depressive symptoms (i.e., GDS).

However, the cross-sectional design of our study is a limitation as it prevents us from assessing the links between baseline levels and changes over time in depressive symptoms and neuroimaging biomarkers. Moreover, the threshold we used to define “subclinical” depressive symptoms was not validated; further studies might allow researchers to select the specific threshold for “clinically relevant” subclinical symptoms. In addition, these symptoms were assessed using a self-report questionnaire; as it is subjective, the measure could be biased by the subject’s honesty, awareness and introspective ability. Further investigations are also needed to clarify the involvement of tau pathology, and of physiological mechanisms related to stress and/or inflammation (e.g., cortisol, cytokines), in the relationship between subclinical depressive symptoms and their brain substrates (especially the hippocampus). In addition, assessing the brain substrates of the subdimensions of depressive symptoms would help specify which of these symptoms are most relevant for future targeted intervention; repetitive negative thinking for instance has been proposed as a risk factor for AD and has been associated with structural alterations in some brain regions identified in our study [82, 83]. In this context, mental training through meditation practice—targeting emotional and attentional regulation and stress reduction—could be a promising way to alleviate depressive symptoms and their adverse factors in ageing. Post-intervention data in Age-Well will be used to address this question [27].

## Conclusion

In conclusion, the presence of subclinical depressive symptoms in CU older adults was associated with brain structural and functional changes in regions mainly belonging to the frontolimbic network, some of which are particularly sensitive to AD. Notably, neurodegeneration markers overlapped in the hippocampus, which alteration may underlie the links between subclinical depressive symptoms in ageing and the risk of clinical depressive disorder and AD dementia. The lack of positive association with amyloid deposition indicates that this link may not involve amyloid deposition, but rather neurodegeneration in partially common brain regions, suggesting that, at this subclinical stage, depressive symptoms is a risk factor for AD rather than a prodromal manifestation of the disease.

## Supplementary information

Supplementary Data
