# Supplementary material for: Depressive symptoms in cognitively unimpaired older adults are associated with lower structural and functional integrity in a frontolimbic network
Source: Mol Psychiatry. 2022 Oct 18;27(12):5086–95. doi: 10.1038/s41380-022-01772-8 (PMC9763117; doi:10.1038/s41380-022-01772-8)
Supplement: Supplementary file 1 — Supplementary Data [file 41380_2022_1772_MOESM1_ESM.docx]

**Supplementary Data**

1. **Supplementary Materials**

**Supplementary Material 1.** Neuroimaging procedures of the primary cohort Age-Well.

**Supplementary Material 2.** Neurodegeneration patterns in Alzheimer’s disease obtained from the IMAP+ cohort.

# Supplementary Material 3. Psychoaffective assessments in the Age-Well cohort.

1. **Supplementary Tables**

**Supplementary Table 1.** Sample size for each neuroimaging modality according to groups and cohorts.

**Supplementary Table 2.** Participants’ characteristics and group comparisons between cohorts.

**Supplementary Table 3.** Detailed statistics of significant neuroimaging clusters.

**Supplementary Table 4.** Relationships between the severity of subclinical depressive symptoms and the levels of brain alterations in both cohorts.

**Supplementary Table 5.** Between-group comparisons of psychoaffective measures in the Age-Well cohort.

1. **Supplementary Figures**

**Supplementary Figure 1.** Results of the voxel-wise between-group differences in myelin integrity of the white matter (radial kurtosis) in Age-Well.

**Supplementary Figure 2.** Results of the voxel-wise between-group differences in glucose metabolism in ADNI with and without partial volume effects (PVE) correction.

1. **References**
2. **Supplementary Materials**

# Supplementary Material 1. Neuroimaging procedures of the primary cohort Age-Well

# MRI data

## T1-weighted images

A high-resolution T1-weighted structural image was acquired using a 3D fast-field echo sequence (3D-T1-FFE sagittal, repetition time = 7.1 ms, echo time = 3.3 ms, flip angle = 6°, 180 slices with no gap, slice thickness = 1mm, field of view = 256x256 mm², in-plane resolution = 1x1x1 mm^3^). T1-weighted images were segmented using FLAIR images (3D-IR sagittal, TR/TE/TI = 4800/272/1650 ms, flip angle = 40°, 180 slices with no gap, slice thickness = 1 mm, field of view = 250x250 mm^2^, in-plane resolution = 0.98x0.98 mm^2^), spatially normalized to the Montreal Neurological Institute (MNI) template, modulated using the SPM12 segmentation procedure (<http://www.fil.ion.ucl.ac.uk>) and smoothed with an 8 mm full-width at half-maximum (FWHM) Gaussian filter. Images were then masked to exclude non-grey matter voxels from the analyses.

## DKI images

An echo-planar imaging/spin echo diffusion weighted sequence (DKI) was performed at multiple shells: 3b-values (0, 1000, 2000 s/mm^2^) (axial, 30 directions, repetition time = 6100 ms, echo time = 101 ms, field of view = 216 × 216 mm^2^, 48 slices, voxel size = 2.7 × 2.7 × 2.7 mm^3^) and additional blips images with b = 0 s/mm2 (number of signal averages = 9) were acquired in reverse phase encoding direction for susceptibility distortion. DKI images were corrected for susceptibility, eddy current distortions and subject motion using Functional Magnetic Resonance of the Brain (FMRIB) diffusion toolbox (FSL 5.0.9, <http://www.fmrib.ox.ac.uk/fsl>). Then, DKI data were processed using Matlab R2012b (MathWorks, Natick, Massachusetts) and the Diffusional Kurtosis Estimator software (DKE: Version 2.6; <http://nitrc.org/projects/dke>) for estimating the diffusional kurtosis tensor [1]. Images were smoothed with a 3.375 × 3.375 × 3.375 mm FWHM Gaussian filter to reduce the impact of noise and misregistration. Mean kurtosis, radial kurtosis and axial kurtosis parameters maps, reflecting white-matter microstructural, myelin and axonal integrity respectively [2, 3], were extracted from the diffusional kurtosis estimator. These maps were then coregistered to their corresponding T1-weighted anatomical MRI and normalized to the MNI template by applying the deformation parameters from the corresponding T1-weighted MRI. Finally, the warped DKI maps were smoothed with a 6.7 x 6.7 x 6.7 mm^3^ FWHM Gaussian kernel, so that the final smoothness of DKI images was the same as anatomical MRI images (8 x 8 x 8 mm^3^). Images were then masked to exclude non-white matter voxels from the analyses.

# PET imaging

Florbetapir- and FDG-PET scans were acquired in two separate sessions with a resolution of 3.76 × 3.76 × 4.9 mm^3^ (field of view = 157 mm). Forty-seven planes were obtained with a voxel size of 1.95 × 1.95 × 3.27 mm^3^. A transmission scan was performed for attenuation correction before the PET acquisition. For the FDG-PET scan, participants (n=92) were fasted for at least 6 hours before scanning. After a 30-min resting period in a quiet and dark environment, 180 MBq of 18F-fluorodeoxyglucose were intravenously injected as a bolus. A 10-min PET acquisition scan began 50 min after injection. For the Florbetapir-PET scan, each participant underwent a 10 min PET scan beginning 50 min after the intravenous injection of ~4MBq/Kg of Florbetapir, reflecting brain amyloid burden. PET images were coregistered on their corresponding T1-weighted MRI, voxel-wise corrected for partial volume effects (PVE) using the three-compartmental voxel-wise Müller-Gärtner method [4], and were then normalized to the MNI template using deformation parameters derived from the anatomical MRI. Resulting images were scaled using cerebellar grey matter as a reference. A smoothing kernel of 10 mm Gaussian filter was applied and images were masked to exclude non-grey matter voxels from the analyses. PVE-corrected normalized and scaled Florbetapir PET images were also used to extract the individual global cortical amyloid standard uptake value ratio (SUVR) using a predetermined neocortical mask including the entire grey matter, except the cerebellum, occipital and sensory motor cortices, hippocampi, amygdala and basal nuclei [5]. The threshold for amyloid positivity was defined as >0.99, and corresponded to the 99.9th percentile of the neocortical SUVR distribution among 45 healthy young individuals, aged <40 years [6].

# Supplementary Material 2. Neurodegeneration patterns in Alzheimer’s disease obtained from the IMAP+ cohort

The Imagerie Multimodale de la maladie d'Alzheimer à un stade Précoce (IMAP+) study was approved by the local ethics committee (CPP Nord-Ouest III) and registered at <http://clinicaltrials.gov> (nb. NTC01638949). After complete description of the study, written informed consent was obtained from all participants. The full methodology for the cohort recruitment and evaluation was detailed in a previous publication [5]. We selected a total of 56 cognitively impaired amyloid-positive patients including 34 MCI and 22 Alzheimer’s-type dementia patients and 28 amyloid-negative cognitively unimpaired older adults as a control group. Patients and controls were matched on age, sex and education level. The MRI and FDG-PET data from the IMAP+ cohort had the same acquisition and pre-processing procedures as the Age-Well cohort (see **Supplementary Material 1**). Voxel-wise group differences in grey matter volume and glucose metabolism were performed using two sample t-tests in SPM12. Results were evaluated for significance at p_uncorrected_<0.001 and k≥1890 voxels for grey matter volume and p_FWE-corrected_<0.05 and k≥200 voxels for glucose metabolism.

# Supplementary Material 3. Psychoaffective assessments in the Age-Well cohort

# Positive and Negative Affect Schedule (PANAS)

The 20-item PANAS includes 20 words that describe different feelings and emotions resulting in two subscales, one measuring positive affect (scores from 10 to 50) and the other measuring negative affect (scores from 10 to 50) [7]. Each item is rated on a 5-point likert-type scale, ranging from 1 (*very slightly or not at all*) to 5 (*extremely*), to indicate the extent to which the respondent has felt this way at the present moment. Higher scores indicate greater positive or negative affect.

# Rumination Response Scale (RRS) – Brooding Subscale

Ruminative brooding assessment was obtained from the full 22-item RRS, with scores from the 5-item brooding subscale (scores from 5 to 20) [8]. Each item is rated on a 4-point likert-type scale, ranging from 1 (*almost never*) to 4 (*almost always*). Higher scores indicate greater ruminative brooding.

# Drexel Defusion Scale (DDS)

The DDS consists of 10 items that evaluate the extent to which individuals can defuse from difficult internal experiences (i.e. their ability to achieve psychological distance from one’s thoughts and feelings) in a specific situation (scores from 0 to 50) [9]. Each item is rated on a 6-point likert-type scale, ranging from 0 (*not at all*) to 5 (*very much*). Higher scores indicate greater psychological defusion.

# Emotion Regulation Questionnaire (ERQ)

The ERQ consists of 10 items that evaluate the tendency to regulate emotions through two strategies, resulting in two distinct subscales: cognitive reappraisal (i.e. changing one's way of thinking in an emotional situation, scores from 6 to 42) or expressive suppression (i.e. inhibiting one’s expressive behaviour in an emotional situation, scores from 4 to 28) [10]. Each item is rated on a 7-point likert-type scale, ranging from 1 (*strongly disagree*) to 7 (*strongly agree*). Higher scores indicate greater cognitive reappraisal or expressive suppression.

1. **Supplementary Tables**

# Supplementary Table 1. Sample size for each neuroimaging modality according to groups and cohorts.

| ***N*** | **NoDepS Group** | **DepS Group** | **Total Group** |
| --- | --- | --- | --- |
| **Age-Well** | 58 | 77 | 135 |
| **Structural MRI**  Grey Matter Volume | 58 | 77 | 135 |
| **DKI**  White Matter Integrity | 58 | 76 | 134 |
| **FDG-PET**  Glucose Metabolism | 39 | 53 | 92 |
| **Florbetapir-PET**  Amyloid Deposition | 58 | 76 | 134 |
| ***N*** | **NoDepS Group** | **DepS Group** | **Total Group** |
| **ADNI** | 118 | 134 | 252 |
| **Structural MRI**  Grey Matter Volume | 118 | 134 | 252 |
| **FDG-PET**  Glucose Metabolism | 118 | 134 | 252 |
| **Florbetapir-PET**  Amyloid Deposition | 117 | 134 | 251 |

Abbreviations: *N* Sample size, *NoDepS* Group without depressive symptoms, *DepS* Group with subclinical depressive symptoms, *MRI* Magnetic Resonance Imaging, *DKI* Diffusion Kurtosis Imaging, *FDG* ^18^F-fluorodeoxyglucose, *PET* Positron Emission Tomography.

# Supplementary Table 2. Participants’ characteristics and group comparisons between cohorts.

| **NoDepS Groups** | **Age-Well** | **ADNI** | **Between-group comparisons** | | |
| --- | --- | --- | --- | --- | --- |
|  |  |  | ***p-value*** | ***t or χ²***  ***value*** | ***Mean difference***  ***[95% CI]*** |
| ***N* (%)** | 58 (43) | 118 (47) |  |  |  |
| **Demographic data** |  |  |  |  |  |
| Gender: Female *N* (%) | 28 (48.27) | 65 (55.08) | 0.49 | 0.48 |  |
| Age, years (Range) | 69.41 ± 3.91 (64-83) | 73.35 ± 5.69 (63-85) | **<0.001** | **4.74** | **3.93 [2.30 to 5.57]** |
| Education, years (Range) | 13.12 ± 3.17 (7-22) | 16.50 ± 2.59 (8-20) | **<0.001** | **7.53** | **3.38 [2.49 to 4.26]** |
| Florbetapir SUVR (Range) | 0.96 ± 0.21 (0.73-1.76) | 1.21 ± 0.37 (0.76-2.55) | **<0.001** | **4.69** | **0.25 [0.14 to 0.35]** |
| Amyloid positive *N* (%) | 10 (17.24) | 69 (58.47) | **<0.001** | **25.08** |  |
| APOEε4 Carriers *N* (%) | 15 (25.86) | 39 (33.05) | 0.57 | 0.33 |  |
| **Global cognition** |  |  |  |  |  |
| MMSE (Range) | 28.93 ± 0.95 (26-30) | 28.99 ± 1.16 (25-30) | 0.73 | 0.34 | 0.06 [-0.29 to 0.41] |
| **Psychoaffective variables** |  |  |  |  |  |
| GDS (Range) | 0.00 ± 0.00 (0-0) | 0.00 ± 0.00 (0-0) | NA | NA | NA |
| STAI-B (Range) | 31.60 ± 6.18 (20-51) | NC | NA | NA | NA |
| **DepS Groups** | **Age-Well** | **ADNI** | **Between-group comparisons** | | |
|  |  |  | ***p-value*** | ***t or χ²***  ***value*** | ***Mean difference***  ***[95% CI]*** |
| ***N* (%)** | 77 (57) | 134 (53) |  |  |  |
| **Demographic data** |  |  |  |  |  |
| Gender: Female *N* (%) | 55 (71.43) | 78 (58.20) | 0.08 | 3.12 |  |
| Age, years (Range) | 68.45 ± 3.63 (65-79) | 73.66 ± 6.32 (59-95) | **<0.001** | **6.63** | **5.21 [3.66 to 6.76]** |
| Education, years (Range) | 13.18 ± 3.04 (7-20) | 16.71 ± 2.35 (12-20) | **<0.001** | **9.42** | **3.53 [2.79 to 4.27]** |
| Florbetapir SUVR (Range) | 0.98 ± 0.20 (0.72-1.73) | 1.13 ± 0.36 (0.74-3.24) | **<0.001** | **3.25** | **0.15 [0.06 to 0.24]** |
| Amyloid positive *N* (%) | 20 (26.31) | 63 (47.01) | **0.01** | **7.85** |  |
| APOEε4 Carriers *N* (%) | 21 (27.27) | 33 (24.63) | 0.45 | 0.57 |  |
| **Global cognition** |  |  |  |  |  |
| MMSE (Range) | 29.11 ± 1.09 (26-30) | 28.96 ± 1.26 (24-30) | 0.39 | -0.87 | -0.15 [-0.49 to 0.19] |
| **Psychoaffective variables** |  |  |  |  |  |
| GDS (Range) | 2.25 ± 1.77 (1-11) | 1.78 ± 1.11 (1-6) | **0.02** | **-2.37** | **-0.47 [-0.86 to -0.08]** |
| STAI-B (Range) | 36.82 ± 6.74 (24-54) | NC | NA | NA | NA |

Abbreviations: *N* Sample size, *NoDepS* Group without depressive symptoms, *DepS* Group with subclinical depressive symptoms, *SUVR* Standard Uptake Value Ratio, *MMSE* Mini-Mental Sate Examination, *GDS* Geriatric Depression Scale, *STAI-B* State-Trait Anxiety Inventory form Y-B, *NC* Not collected, *NA* Not applicable.

**Supplementary Table 3.** Detailed statistics of significant neuroimaging clusters.

| **Neuroimaging Modality and Brain Region** | **Cluster-level** | | | **Peak-level** | | |
| --- | --- | --- | --- | --- | --- | --- |
|  | Nb of Voxels | mm^3^ | Minimum Cluster Size | MNI Coordinates  x y z | p_uncorrected_ | T-value |
| **Age-Well** |  |  |  |  |  |  |
| **Structural MRI**  Grey Matter Volume |  |  |  |  |  |  |
| R Hippocampus | 245 | 827 | 242 | 38 -30 -9 | <0.001 | 3.69 |
| **DKI**  White Matter Integrity |  |  |  |  |  |  |
| **Mean Kurtosis** |  |  |  |  |  |  |
| R Fornix, R Corticospinal Fasciculus, R Inferior Longitudinal Fasciculus, R Internal Capsule, R Cortico-Ponto-Cerebellar Fasciculus | 829 | 2798 | 519 | 30 -32 2 | 0.001 | 3.24 |
| L Posterior Cingulum, B Posterior Corpus Callosum (Splenium Part) | 639 | 2157 | 519 | -10 -48 18 | <0.001 | 3.81 |
| **Radial Kurtosis** |  |  |  |  |  |  |
| B Posterior Cingulum, B Posterior Corpus Callosum (Splenium Part) | 1465 | 4944 | 784 | -10 -46 18 | <0.001 | 3.79 |
| **FDG-PET**  Glucose metabolism | NS | NS | NS | NS | NS | NS |
| **Florbetapir-PET**  Amyloid deposition | NS | NS | NS | NS | NS | NS |
| **ADNI** |  |  |  |  |  |  |
| **Structural MRI**  Grey matter volume |  |  |  |  |  |  |
| L Hippocampus | 248 | 837 | 205 | -26 -18 -15 | <0.001 | 3.56 |
| **FDG-PET**  Glucose metabolism |  |  |  |  |  |  |
| B Inferior Frontal Cortex, B Temporal Cortex (Superior and Middle Parts), L Occipital Cortex (Fusiform and Lingual Parts), L Hippocampal/Parahippocampal Regions, L Amygdala, B Prefrontal Cortex (Medial and Dorsolateral Parts), B Anterior Cingulate Cortex, B Insula, R Temporoparietal Cortex, B Putamen and Globus Pallidus | 24841 | 83838 | 1554 | 56 2 3 | <0.001 | 4.40 |
| R Occipital Cortex (Fusiform and Lingual regions), R Hippocampal/Parahippocampal Regions, R Precuneus/Posterior Cingulate Cortex | 2440 | 8235 | 1554 | 28 -60 -4 | <0.001 | 3.63 |
| **Florbetapir-PET**  Amyloid deposition | NS | NS | NS | NS | NS | NS |

Results were evaluated for significance at p_uncorrected_<0.005 combined with a minimum cluster size determined by Monte‐Carlo simulations using the AFNI’s 3dClustSim program to achieve a corrected statistical significance of p<0.05. The first region mentioned corresponds to the statistical peak, and the others regions listed compose the rest of each cluster. Abbreviations: *MRI* Magnetic Resonance Imaging, *DKI* Diffusion Kurtosis Imaging, *FDG* ^18^F-fluorodeoxyglucose, *PET* Positron Emission Tomography, *R* Right, *L* Left, *B* Bilateral, *NS* Not significant.

**Supplementary Table 4.** Relationships between the severity of subclinical depressive symptoms and the levels of brain alterations in both cohorts.

| **Spearman’s Partial Correlations**  **with Subclinical Depressive Symptoms (GDS score)** | **DepS Group** | **Total Group** |
| --- | --- | --- |
| **Age-Well** | **(n=77)** | **(n=135)** |
| **Structural MRI** Grey Matter Volume | 0.180 (0.128) | **-0.211 (0.016)** |
| **DKI** White Matter Integrity |  |  |
| *Mean Kurtosis* | 0.145 (0.225) | **-0.232 (0.008)** |
| *Radial Kurtosis* | 0.024 (0.845) | **-0.236 (0.007)** |
| **ADNI** | **(n=134)** | **(n=252)** |
| **Structural MRI** Grey Matter Volume | -0.095 (0.281) | **-0.251 (<0.001)** |
| **FDG-PET** Glucose metabolism | -0.021 (0.814) | **-0.277 (<0.001)** |

Values indicate rho (p) values of the Spearman’s partial correlations between the severity of subclinical depressive symptoms and the mean neuroimaging values extracted from the voxel-wise clusters highlighted in the main previous analyses – within the DepS group and the entire samples, for both cohorts. All analyses were adjusted for age, sex, education, as well as anxiety symptoms (only for Age-Well). Values in bold correspond to significant p-values (p<0.05). Abbreviations: *DepS* Group with subclinical depressive symptoms, *GDS* Geriatric Depression Scale, *MRI* Magnetic Resonance Imaging, *DKI* Diffusion Kurtosis Imaging, *FDG* ^18^F-fluorodeoxyglucose, *PET* Positron Emission Tomography.

**Supplementary Table 5.** Between-group comparisons of psychoaffective measures in the Age-Well cohort

| **Age-Well – Primary cohort**  **(N=135)** | **NoDepS**  **Group** | **DepS**  **Group** | **Between-group comparisons** | |
| --- | --- | --- | --- | --- |
|  |  |  | ***p-value*** | ***F value*** |
| ***N* (%)** | 58 (43) | 77 (57) |  |  |
| **Psychoaffective variables** |  |  |  |  |
| PANAS Positive (Range) | 33.76 ± 4.85 (22-45) | 32.81 ± 4.55 (17-41) | 0.170 | 1.90 |
| PANAS Negative (Range) | 11.07 ± 1.72 (10-17) | 12.26 ± 2.93 (10-25) | **0.017** | **5.82** |
| RRS Brooding (Range) | 7.62 ± 1.94 (5-15) | 8.47 ± 2.47 (5-16) | **0.040** | **4.32** |
| DDS Defusion (Range) | 36.12 ± 5.75 (19-48) | 33.10 ± 5.12 (19-43) | **0.003** | **9.33** |
| ERQ Cognitive Reappraisal (Range) | 30.09 ± 5.74 (15-40) | 29.33 ± 5.67 (6-40) | 0.400 | 0.71 |
| ERQ Expressive Suppression (Range) | 16.74 ± 5.49 (4-28) | 16.40 ± 4.98 (4-28) | 0.865 | 0.03 |

Data are presented as mean ± standard deviation of participants. Between-group differences were assessed using analyses of covariance (ANCOVA) adjusted for age, sex and education. Values in bold correspond to significant p-values (p<0.05). Abbreviations: *N* Sample size, *NoDepS* Group without depressive symptoms, *DepS* Group with subclinical depressive symptoms, *PANAS* Positive and Negative Affect Schedule, *RRS* Rumination Response Scale, *DDS* Drexel Defusion Scale, *ERQ* Emotion Regulation Questionnaire.

1. **Supplementary Figures**


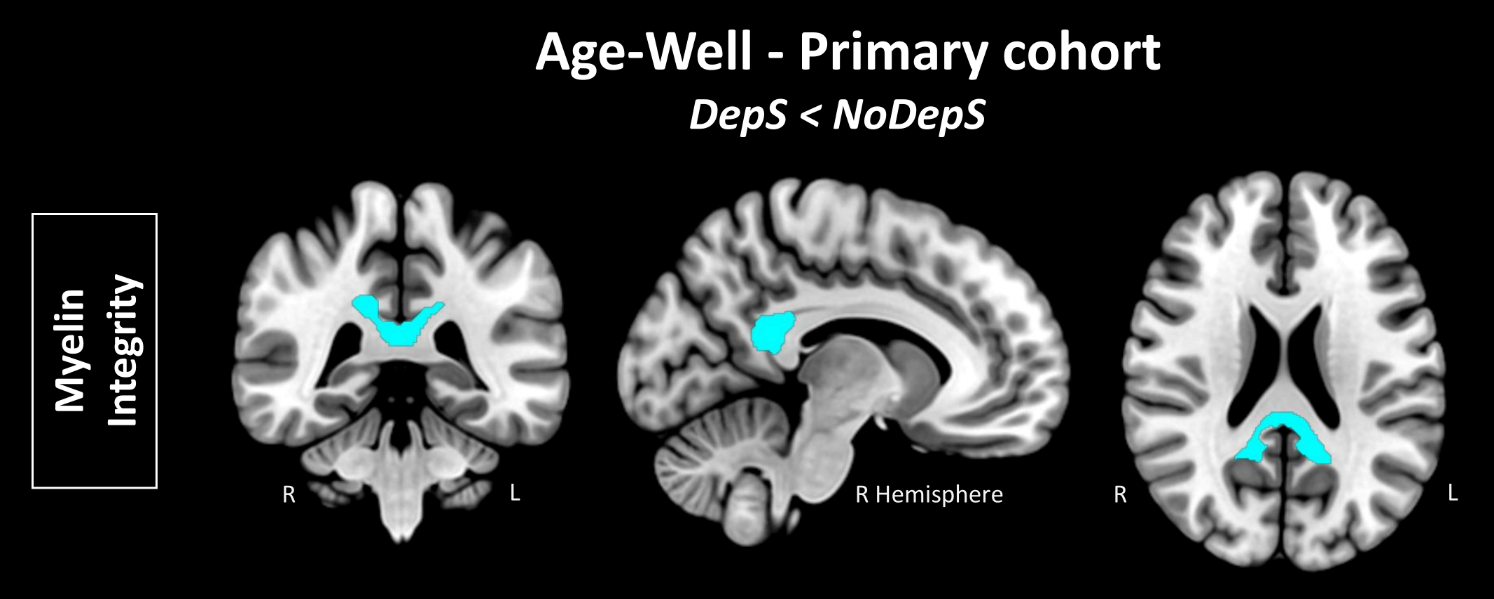


**Supplementary Figure 1.** Results of the voxel-wise between-group differences in myelin integrity of the white matter (radial kurtosis) in Age-Well. Analyses were adjusted for age, sex, education and anxiety symptoms. All results are presented at a p_uncorrected_<0.005 threshold combined with a cluster-level multiple comparisons correction. *DepS* Group with subclinical depressive symptoms, *NoDepS* Group without depressive symptoms, *R* right, *L* Left.


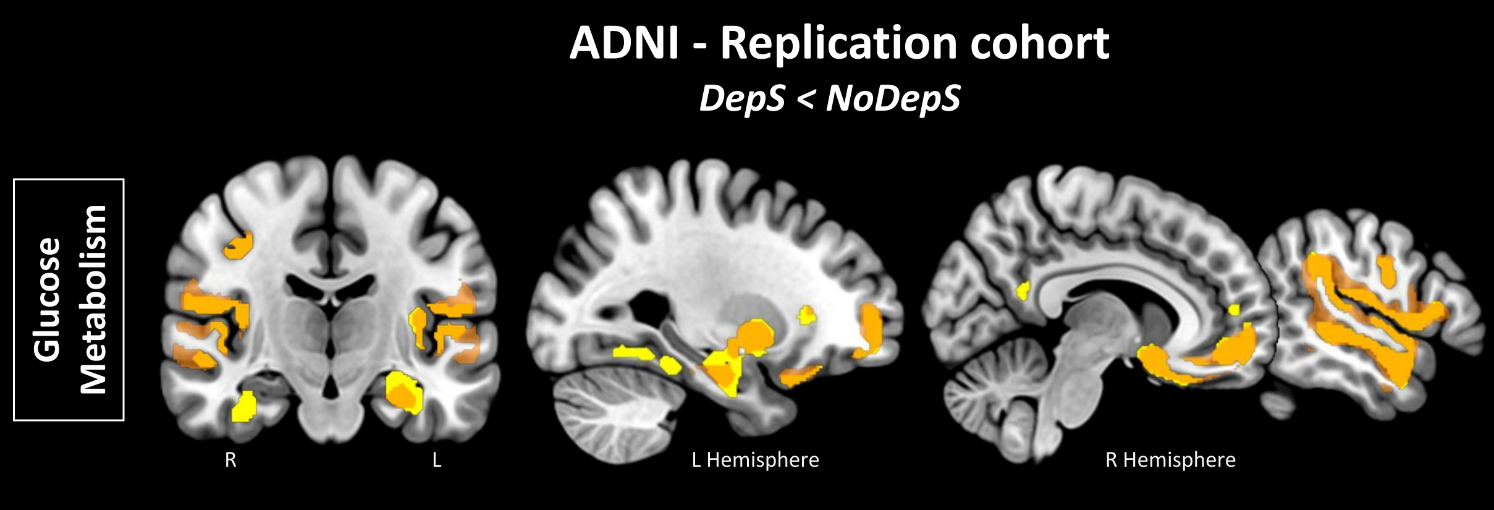


**Supplementary Figure 2.** Results of the voxel-wise between-group differences in glucose metabolism in ADNI with (yellow) and without (orange) partial volume effects (PVE) correction. Analyses were adjusted for age, sex and education. All results are presented at a p_uncorrected_<0.005 threshold combined with a cluster-level multiple comparisons correction. Analyses of between-group comparisons for glucose metabolism in Age-Well and amyloid deposition in both cohorts were not significant with and without PVE correction. *DepS* Group with subclinical depressive symptoms, *NoDepS* Group without depressive symptoms, *R* Right, *L* Left.

1. **References**

1. Tabesh A, Jensen JH, Ardekani BA, Helpern JA. Estimation of tensors and tensor-derived measures in diffusional kurtosis imaging. Magnetic Resonance in Medicine. 2011;65:823–836.

2. Falangola MF, Jensen JH, Babb JS, Hu C, Castellanos FX, Martino AD, et al. Age-related non-Gaussian diffusion patterns in the prefrontal brain. Journal of Magnetic Resonance Imaging. 2008;28:1345–1350.

3. Falangola MF, Jensen JH, Tabesh A, Hu C, Deardorff RL, Babb JS, et al. Non-Gaussian diffusion MRI assessment of brain microstructure in mild cognitive impairment and Alzheimer’s disease. Magn Reson Imaging. 2013;31:840–846.

4. Müller-Gärtner HW, Links JM, Prince JL, Bryan RN, McVeigh E, Leal JP, et al. Measurement of Radiotracer Concentration in Brain Gray Matter Using Positron Emission Tomography: MRI-Based Correction for Partial Volume Effects: Journal of Cerebral Blood Flow & Metabolism. 2016. 29 June 2016. https://doi.org/10.1038/jcbfm.1992.81.

5. La Joie R, Perrotin A, de La Sayette V, Egret S, Doeuvre L, Belliard S, et al. Hippocampal subfield volumetry in mild cognitive impairment, Alzheimer’s disease and semantic dementia. Neuroimage Clin. 2013;3:155–162.

6. André C, Rehel S, Kuhn E, Landeau B, Moulinet I, Touron E, et al. Association of Sleep-Disordered Breathing With Alzheimer Disease Biomarkers in Community-Dwelling Older Adults: A Secondary Analysis of a Randomized Clinical Trial. JAMA Neurol. 2020;77:716–724.

7. Watson D, Clark LA, Tellegen A. Development and validation of brief measures of positive and negative affect: the PANAS scales. J Pers Soc Psychol. 1988;54:1063–1070.

8. Treynor W, Gonzalez R, Nolen-Hoeksema S. Rumination Reconsidered: A Psychometric Analysis. Cognitive Therapy and Research. 2003;27:247–259.

9. Forman EM, Herbert JD, Juarascio AS, Yeomans PD, Zebell JA, Goetter EM, et al. The Drexel defusion scale: A new measure of experiential distancing. Journal of Contextual Behavioral Science. 2012;1:55–65.

10. Gross JJ, John OP. Individual differences in two emotion regulation processes: implications for affect, relationships, and well-being. J Pers Soc Psychol. 2003;85:348–362.
